# A global view of substrate phosphorylation and dephosphorylation during budding yeast mitotic exit

**DOI:** 10.15698/mic2018.08.644

**Published:** 2018-07-25

**Authors:** Sandra A. Touati, Frank Uhlmann

**Affiliations:** 1 Chromosome Segregation Laboratory, The Francis Crick Institute, London, UK.

**Keywords:** cell cycle, kinases, mitosis, mitotic exit, phosphatases, phosphoproteomics

## Abstract

The cell cycle is the process by which a cell duplicates its DNA during S-phase and divides its chromosomes during M-phase, creating two genetically identical daughter cells. Cell cycle events are ordered by synthesis and degradation of key cell regulators and by phosphorylation and dephosphorylation of numerous substrates. Phosphorylation can alter the activity, interactions or subcellular localization of a protein. A substrate’s phosphorylation status is the readout of competing activities of kinases and phosphatases that target each of its phosphorylation sites. In our recent study (EMBO J. 37, e98745), we performed time-resolved global phosphoproteome analysis of a period during the cell cycle known as mitotic exit. During this time, numerous cell biological events happen in fast succession but in strict order. First, at the metaphase to anaphase transition, the mitotic spindle elongates to pull maximally condensed chromosomes to opposite cell halves. Shortly after that, spindles disassemble and chromosomes decondense, before finally cell division is completed by cytokinesis. Our time-resolved phosphoproteome analysis of this period in budding yeast provided a survey of the principles of phosphoregulation used to order these events.

## INTRODUCTION

Cyclin-dependent kinase (Cdk) is the master cell cycle regulator. In complex with its regulatory cyclin subunits, Cdk phosphorylates numerous substrates at distinct cell cycle stages. Specificity arises from the Cdk active site that recognizes the phosphorylation site. Cdk is a proline directed serine/threonine kinase, meaning that its substrates are minimally defined by an (S/T)P motif. A basic residue at the +3 position is preferred, giving rise to what is known as the full Cdk consensus motif (S/T)Px(K/R). In addition, the cyclins add substrate specificity by using a hydrophobic patch that recognizes substrate docking motifs. These docking motifs can be found on substrates at a distance from the phosphorylation site. During the budding yeast cell cycle, nine different cyclins are synthesized during progression from G1 until mitosis, conferring substrate specificity through the different cell cycle stages. In addition to their distinct specificities, the different cyclin-Cdk complexes build up increasing overall Cdk activity as cells progress from G1 towards mitosis. Cdk activity is low during G1, increases during S phase and culminates in mitosis. As the peak in Cdk activity is reached in metaphase, the anaphase promoting complex/cyclosome (APC/C), a large ubiquitin ligase complex, is activated. First in conjunction with its coactivator Cdc20, APC^Cdc20^ and later with Cdh1, APC^Cdh1^, they cause gradual cyclin destruction. S-phase cyclins are degraded first, followed by M-phase cyclins. This allows the timely dephosphorylation of Cdk substrates during the course of mitotic exit.

Phosphatases that counteract Cdk phosphorylation are essential during mitotic exit to bring about ordered substrate dephosphorylation. The changing kinase-to-phosphatase ratio instructs each substrate to respond with dephosphorylation at their respective times. This guarantees that substrates controlling spindle elongation are dephosphorylated before substrates that ultimately promote cytokinesis. While Cdk phosphorylation and dephosphorylation provides a crucial level of control, numerous additional kinases are present and active during mitotic exit. These also phosphorylate serine and threonine residues, albeit within their own consensus motif surroundings. In budding yeast, these include Cdc5 (Polo-like kinase), Ipl1 (Aurora kinase), Dbf2-Mob1 and Cbk1-Mob2 (two members of the Nuclear Dbf2-Related (NDR) kinase family). Mutations in any of these lead to chromosome segregation failures, suggesting that mitotic exit is not only governed by Cdk substrate dephosphorylation, but that phosphorylation events controlled by these additional kinases must also be considered. To gain a global overview of phosphoregulation during mitotic exit, we wanted to measure the timing of as many as possible phosphorylation and dephosphorylation events that take place. With this, we wanted to address three questions: How does the phosphorylation status of each individual phosphosite develop over the course of mitotic exit? How do multiple phosphosites on the same protein respond with respect to each other? Can we identify phosphosite features that predict its behavior during mitotic exit?

To survey phosphoproteome dynamics during budding yeast mitotic exit, we performed a time-resolved phosphoproteome analysis. We synchronized yeast cultures by depleting the APC coactivator Cdc20 under control of a galactose-inducible promoter by galactose withdrawal. This leads to a uniform cell arrest in metaphase. Galactose readdition induces Cdc20 and starts synchronous progression through mitotic exit. Ten samples taken at five minute intervals covered the mitotic exit stages from metaphase to cytokinesis and return to G1. We used Stable Isotope Labelling with Amino acids in Cell culture (SILAC) to generate a metaphase culture that was added as a common internal comparison to all ten samples. Following cell breakage and phosphopeptide enrichment, we used LC-MS/MS mass spectrometry to identify and quantify phosphosites, 3456 of which could be followed throughout the ten timepoints. 1101 unique proteins carried at least one phosphosite, corresponding to 20% of the budding yeast proteome. Of those, 346 proteins contained at least one phosphosite that changed in abundance during mitotic exit.

**Figure 1:**
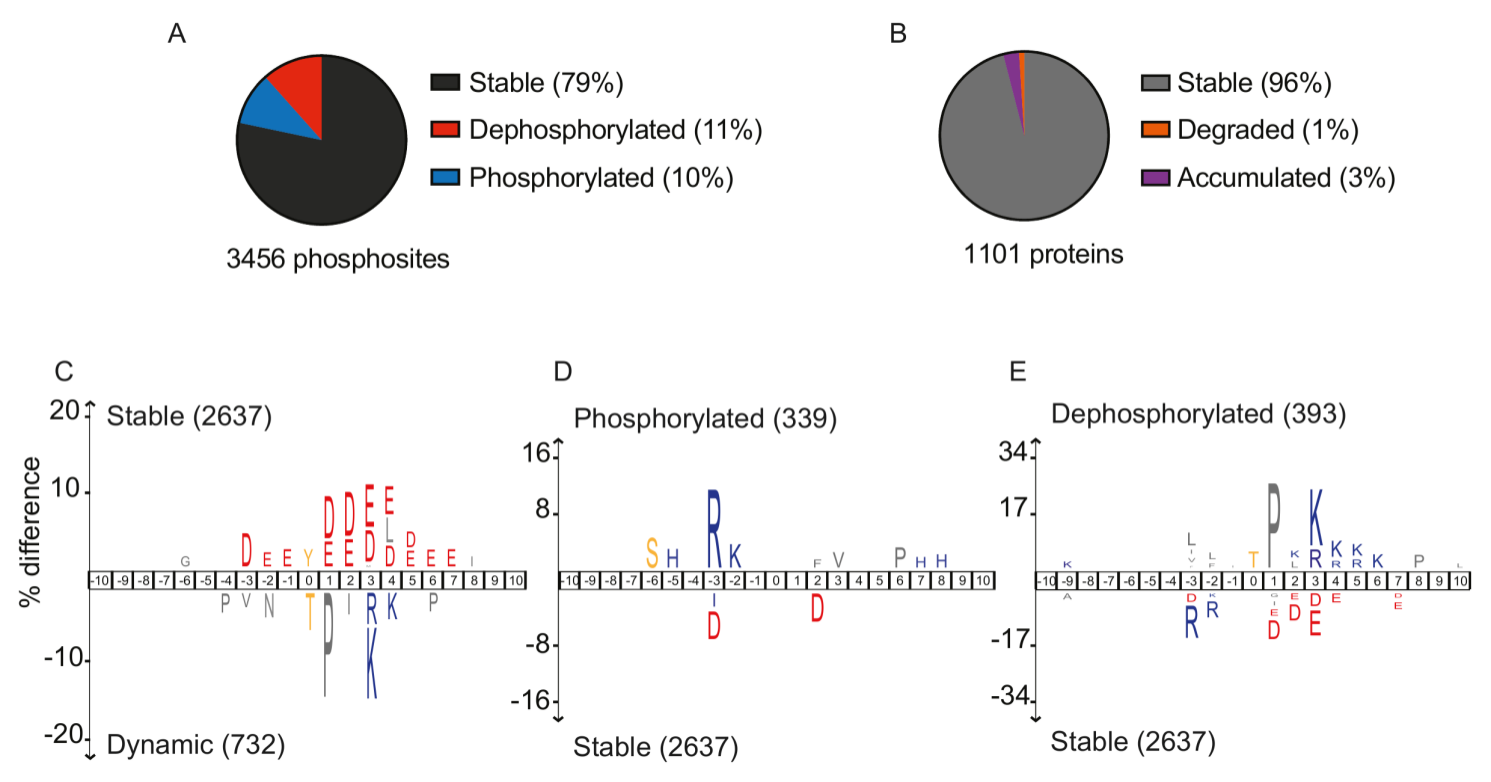
FIGURE 1: Dynamic phosphosite behavior during mitotic exit correlates with sequence surroundings. **(A)** Fractions of stable (black), dephosphorylated (red) and phosphorylated (blue) phosphosites during mitotic exit. **(B)** Fractions of stable (grey), degraded (orange) and accumulated (purple) proteins. **(C-E)** IceLogo motif analyses to reveal sequence elements enriched around phosphorylation sites. These analyses compared **(C)** dynamic versus stable, **(D)** phosphorylated versus stable and **(E)** dephosphorylated versus stable phosphosites. The phosphorylated residues are at position ‘0’. Larger letter size indicates increasing enrichment, cutoff for enrichment detection was *p* = 0.01.

Looking at overall abundance changes, 79% of phosphosites did not change during mitotic exit. Their constitutive phosphorylation might maintain protein conformation, or phosphorylation changes might occur at other cell cycle stages or in response to other stimuli. 11% of all phosphosites were dephosphorylated during mitotic exit, while 10% gained phosphorylation (Figure 1A). As Cdk activity is downregulated during mitotic exit, and Polo and Aurora kinases are degraded towards the end, mitotic exit was thought to be a period of overall protein dephosphorylation. We were therefore surprised to find an almost equal number of sites that gained phosphorylation during mitotic exit. The time resolution of our experiment afforded further insight. 86 proteins carried phosphosites that first increased in abundance during anaphase and were then dephosphorylated at later timepoint towards cytokinesis. ‘Dephosphorylation’ in our analysis means reduced phosphosite abundance. This could happen by dephosphorylation or by protein destruction. To gauge the possible contribution of proteolysis to phosphosite loss, we also measured overall protein levels. Only 1% of all detected proteins decreased in abundance during mitotic exit (Figure 1B). While the APC/C is a prominent mitotic exit regulator, this suggests that phosphoregulation affects a far greater number of proteins, compared to proteolysis. We note that our mass spectrometry approach reports on relative phosphosite abundance changes. It does not reveal what fraction of the total protein carries the modification. This important additional information is harder to obtain. Mass spectrometric methods are being developed to address this, which will be a focus of future studies.

For many proteins, we detected modification on more than one residue. In some cases, multiple phosphosites were coregulated, i.e. they decreased, or increased, with similar kinetics. However, there were many cases where different phosphosites on the same protein behaved very differently. 64 proteins contained both dephosphorylated sites as well as sites that gained phosphorylation during mitotic exit. Even when looking at the 65 proteins with more than one phosphosites that were dephosphorylated, we found surprising variability. If we classify these sites into three groups based on their dephosphorylation timing (early, up to 15 minutes; intermediate, 20-30 minutes; late, from 35 minutes), then half of all phosphosites differ in dephosphorylation timing from other sites on the same protein. This illustrates that each phosphosite is subject to its own regulation. This could include priming modifications, as well as differential targeting of individual phosphosites by their respective kinases and phosphatases.

To test how the peptide sequence surrounding each phosphorylation site affects its behavior, we performed three comparative motif analyses, using IceLogo. First, we compared all stable phosphosites with all those that changed either up or down. This revealed a notable enrichment for acidic amino acids (D/E) surrounding stable phosphosites, a feature worth exploring further (Figure 1C). In contrast, dynamic phosphosites were often threonines followed by proline (P) and positively charged amino acids (K/R), characteristics of the full Cdk consensus motif. Next, we specifically compared sites that gain phosphorylation with all stable sites. This showed that positively charged amino acids are enriched at the -2 and -3 positions, which could be rationalized by the sequence preference of late mitotic Aurora and NDR kinases. Negative charges again were characteristic for the stable sites (Figure 1D). Finally, we compared dephosphorylated sites to their stable counterparts. This revealed that threonine residues are preferentially dephosphorylated, suggesting that a threonine-directed phosphatase(s) impacts on substrate dephosphorylation during budding yeast mitotic exit. Hydrophobic amino-acids upstream, a proline in +1 position and additional positive charges in +3, 4, 5 or 6 positions are additional features that are enriched among dephosphorylated sites (Figure 1E). These features partly match the substrate preferences of the most studied mitotic exit phosphatase in budding yeast, Cdc14. This phosphatase accommodates a +1 proline in its active site, and prefers downstream positive charges. However, Cdc14 is a serine-directed phosphatase, threonines produce a steric clash with its active site. PP2A phosphatases in turn show a preference for threonine. PP2A plays important roles during mitotic exit in mammals, its contribution to budding yeast mitotic exit deserves further investigation.

**Figure 2:**
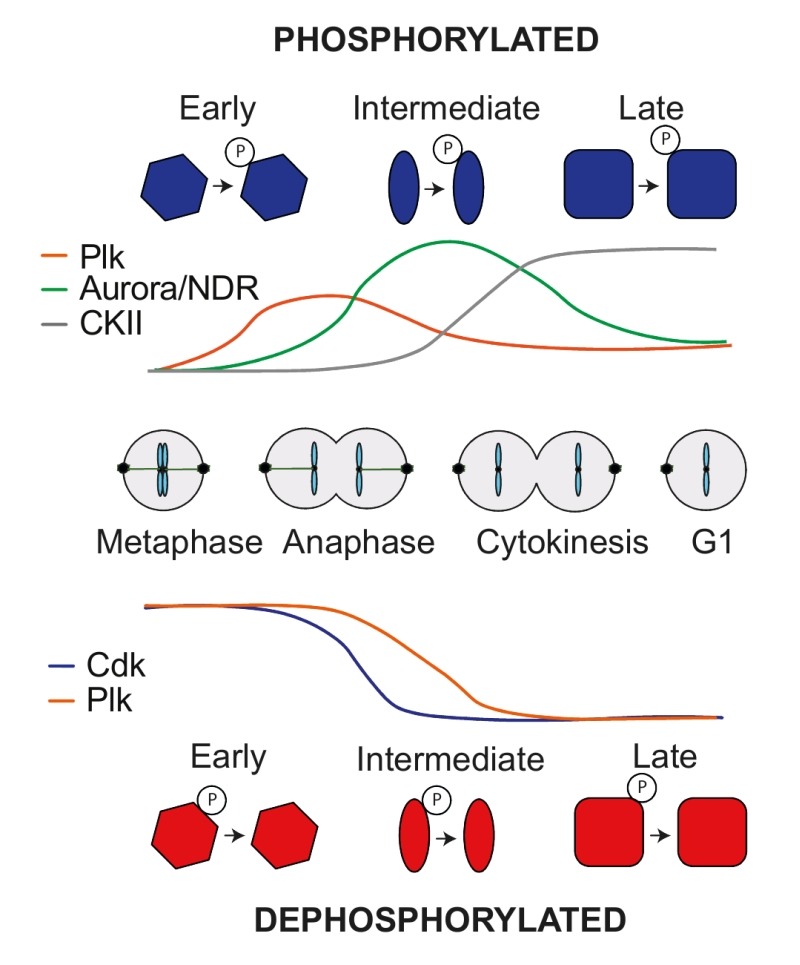
FIGURE 2: Phosphoproteome dynamics during mitotic exit controlled by kinases. Scheme summarizing how kinases impact on both protein phosphorylation and dephosphorylation during mitotic exit. Phosphosites carrying Polo-like kinase consensus motifs are phosphorylated early during mitotic exit, followed by Aurora and NDR family kinases and finally casein kinase II (CKII) consensus motifs. In turn, phosphosites displaying a full Cdk consensus motif are dephosphorylated first, followed by minimal Cdk motifs and finally Polo-like kinase motifs.

While phosphatases are thought to target broad spectrums of substrates, kinases display greater specificity during sequence motif recognition. This allowed us to address the contribution of specific kinases to phosphosite regulation during mitotic exit. Looking at all newly phosphorylated sites, a Polo-like kinase consensus motif (D/E/N)p(S/T) dominated early phosphorylated sites. Those sites often lost phosphorylation again at later times. NDR kinase Rxx(S/T) and Aurora kinase R(R/K)x(S/T) consensus motifs became phosphorylated in a second wave, many of which also declined towards the end of mitotic exit. Finally, CKII kinase consensus motifs Sxx(D/E) were phosphorylated late in anaphase and remained stable until the end of the experiment (Figure 2). Turning to substrate dephosphorylation also revealed kinase signatures. Full Cdk consensus sites (S/T)Px(K/R) were the first to be dephosphorylated in early anaphase, followed by minimal Cdk phosphosites. The majority of Polo consensus motifs started to become dephosphorylated only later (Figure 2). The special status of full Cdk consensus sites became further apparent when looking at the fraction of sites that are dephosphorylated. As we saw above, 11% of all phosphosites are dephosphorylated during mitotic exit. The fraction of dephosphorylated sites that match a minimal Cdk or a Polo consensus sites is also around 11%. Strikingly, over 60% of full Cdk sites are efficiently dephosphorylated during mitotic exit. This ascribes particular predictive power for cell cycle regulation to a full Cdk consensus motif.

Our results highlight the importance of kinases and their dynamic regulation in shaping the phosphorylation landscape during mitotic exit. Phosphomotif identities, distributed amongst the many regulated substrates, confer temporal input to their modification dynamics. The phosphorylation dynamics will be passed on to the biological processes that these substrates control. In the future, it will be important to delineate the contributions of cell cycle phosphatases to phosphoproteome dynamics during eukaryotic cell cycle progression.

